# Smad4 regulates the nuclear translocation of Nkx2-5 in cardiac differentiation

**DOI:** 10.1038/s41598-021-82954-2

**Published:** 2021-02-11

**Authors:** Wenyu Hu, Anqi Dong, Kohei Karasaki, Shota Sogabe, Daiki Okamoto, Masato Saigo, Mari Ishida, Masao Yoshizumi, Hiroki Kokubo

**Affiliations:** 1 Department of Cardiovascular Physiology and Medicine, Graduate School of Biomedical and Health Sciences, Hiroshima University, 1-2-3 Kasumi, Minamiku, Hiroshima 734-8551 Japan; 2 Department of Cardiology, The First Affiliated Hospital of China Medical University, Shenyang, 110001 Liaoning China

**Keywords:** Differentiation, Heart development

## Abstract

Bmp plays an important role in cardiomyocyte differentiation, but the function of Smad4 in Bmp signaling remains elusive. Here, we show that disruption of the *Smad4* gene in cardiac progenitors expressing *Sfrp5* led to embryonic lethality with hypoplastic heart formation. Although the expression of *Nkx2-5* is regulated by Bmp signaling, expression of *Nkx2-5* was weakly detected in the mutant heart. However, the nuclear translocation of Nkx2-5 was impaired. Expression of *CK2* or *PP1*, which could alter the phosphorylation status of the NLS of Nkx2-5, was not affected, but Nkx2-5 was found to bind to Smad4 by co-immunoprecipitation experiments. Introduction of *Smad4* into cells derived from *Smad4* conditional knockout embryonic hearts restored the nuclear localization of Nkx2-5, and exogenous Nkx2-5 failed to translocate into the nucleus of *Smad4*-depleted fibroblasts. These results suggest that Smad4 plays an essential role in cardiomyocyte differentiation by controlling not only transcription but also the nuclear localization of Nkx2-5.

## Introduction

The heart is the first organ to establish a blood circulatory system required for the subsequent development of other organs. Decades of research have revealed that intricate signaling networks, including those involving members of the Wnt, Bmp, and Fgf pathways, play key roles in heart development because perturbation of these secreted factors has been shown to result in a wide range of defects in embryonic and adult hearts^1–5^. Understanding how signaling networks regulate heart development is critical for elucidating mechanisms underlying congenital heart diseases and for developing novel approaches in regenerative cardiovascular medicine.

Bmp signaling is involved in fate decisions of multiple cell lineages of cardiomyocytes through the transcriptional regulation of cardiac-specific transcription factors, such as GATA4, Mef2c, and Nkx2-5^6–13^. Bmp signaling has been reported to directly regulate transcription of the *Nkx2-5* gene, which encodes one of the earliest and most important cardiac transcription factors^14,15^, given the presence of Smad binding sites in the enhancer region of the *Nkx2-5* gene^13^. Smad4 is a common-mediator Smad (Co-Smad). Once the Bmp receptor is activated by ligand binding, Smad1/5/8 is phosphorylated and forms a complex with Smad4. This complex then translocates into the nucleus and regulates the transcription of target genes, suggesting that Smad4 plays a central role in Bmp signaling.

*Smad4*-deficient mouse embryos die by E7.5 due to impaired gastrulation, suggesting that Smad4 is required for mesodermal formation^16^. An analysis of cardiomyocyte-specific *Smad4*-KO mice revealed that Smad4 functions in the proliferation and differentiation of cardiomyocytes, as reflected in the thinner ventricles in both trabecular and compact hearts^17^. However, the function of Smad4 during the process of cardiomyocyte differentiation remains unknown.

Wnt proteins are secreted proteins that play a key role in differentiation during the development of many organs, including the heart^18–20^. In a previous study, we reported that Sfrp5 (a decoy receptor of the Wnt ligand) is a marker of cardiac progenitor cells, which form the first heart field (FHF) and later contribute to the left ventricle (LV), both atria, sinus venous (SV), and a part of the outflow tract (OFT), but not to the right ventricle (RV)^21^. Thus, mice that express Cre in *Sfrp5*-expressing cells could serve as a tool to generate mice that specifically lack the *Smad4* gene in cardiac progenitor cells.

In the present study, we provide evidence that Smad4 forms a complex with Nkx2-5 to control the nuclear localization of Nkx2-5, and that this in turn promotes the differentiation of cardiac progenitors and heart morphogenesis. To examine specific functions of Smad4 in cardiogenesis, we generated a *Smad4*-conditional knock-out (*Smad4*-cKO) model by crossing *Smad4*^*floxed/floxed*^ mice with *Sfrp5*-Cre mice. *Smad4*-cKO mice exhibited severe cardiac hypoplasia with inhibited myocardial cell differentiation due to the down-regulation of *Nkx2-5* expression and loss of nuclear localization of Nkx2-5. These findings suggest that, in addition to a mediator of Bmp signaling, Smad4 could be responsible for cardiac differentiation by binding to Nkx2-5 and regulating its nuclear localization.

## Results

### Smad4 is required for proper cardiogenesis

**Figure 1:**
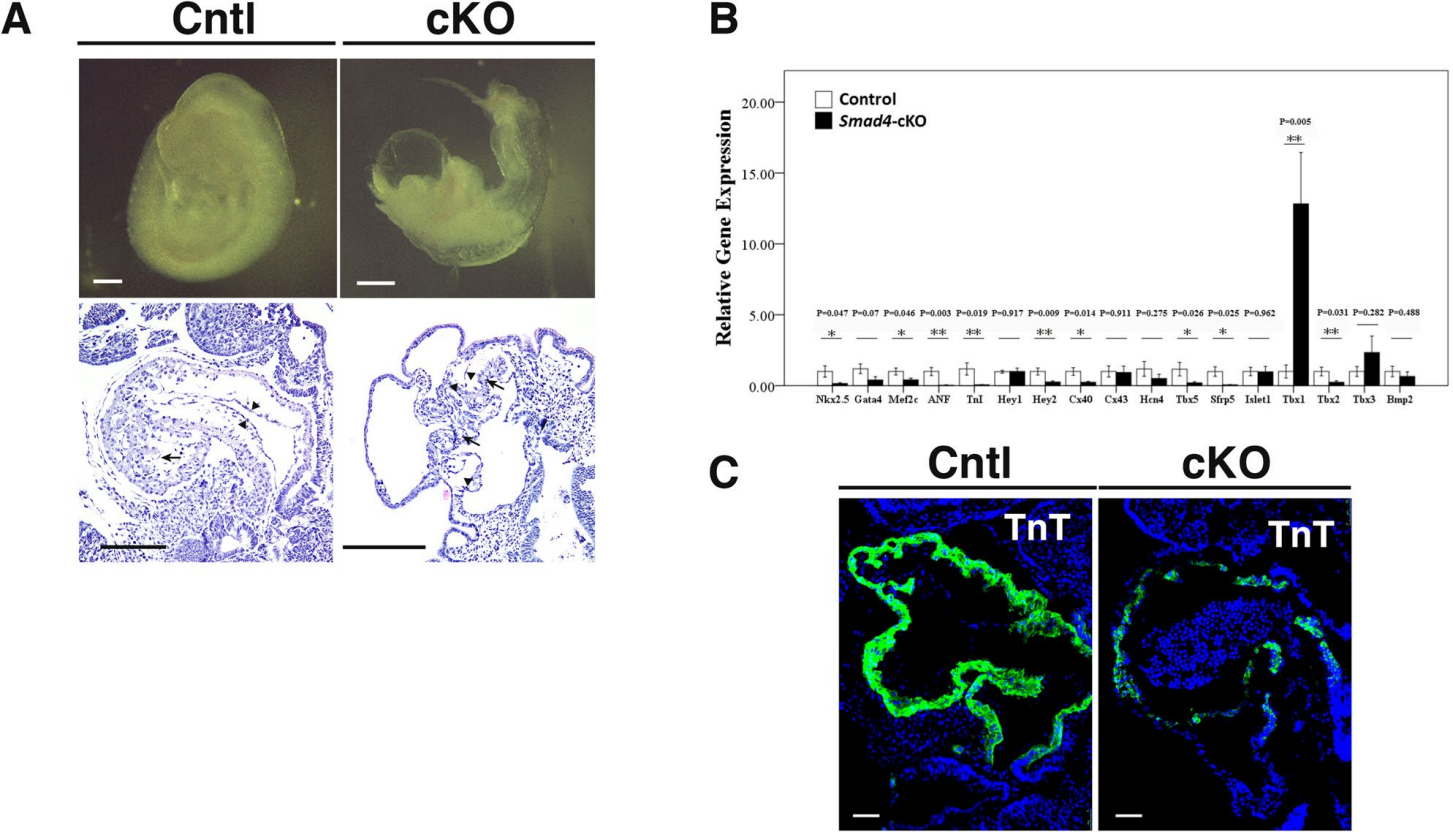
Disruption of the *Smad4* gene in *Sfrp5*-expressing cells leads to hypoplastic heart formation and impaired cardiac differentiation. (**A**,**B**) Gross appearance (**A**) and Hematoxylin and Eosin staining (**B**) of sagittal sections of *Smad4* mutant (*Sfrp5 *^*Cre/*+^*; Smad4 *^*del/del*^) and littermate control (*Sfrp5 *^*Cre/*+^*; Smad4*^*del/*+^) embryos dissected at E9.5. Scale bar = 100 μm. (**C**) Relative gene expression levels of key factors involved in cardiogenesis. n = 9 and 7 for control and *Smad4*-cKO, respectively. Data are presented as mean ± SEM. (**D**) Immunofluorescent staining for cTnT (green) in control and *Smad4* mutant embryos at E9.5. Samples were counterstained with 4′, 6-diamidino-2-phenylindole (DAPI) to visualize nuclei (blue). Scale bar = 50 μm.

In order to explore the function of Smad4 in cardiomyocyte differentiation, we disrupted the *Smad4* gene in *Sfrp5*-expressing cardiac progenitor cells. Specifically, *Sfrp5 *^*Cre/Cre*^*; Smad4 *^*del/*+^ mice were crossed with *Smad4f.*^*/f*^ mice^21^. Compared to the littermate controls (*Sfrp5 *^*Cre/*+^*; Smad4*^*del/*+^), *Smad4*-cKO mice (*Sfrp5 *^*Cre/*+^*; Smad4 *^*del/del*^) were embryonic lethal at around E8.5, with a hypoplastic heart tube and edema of the pericardium (Fig. 1A). The looping process has already begun but stopped in the middle of the process in the mutant hearts (Supplementary Fig. S1). Histological analysis showed that both trabecular and compact myocardial layers in the mutant heart were much thinner than those in the control. This suggests that Smad4 is required for heart development.

Since Bmp signaling regulates many genes involved in myocardial differentiation, we assessed the expression of downstream target genes by RT-PCR to gain insight into the potential cause of the hypoplastic heart in *Smad4*-cKO embryos. Transcription factors required in the myocardial differentiation process, including *Nkx2-5*, *Gata4*, and *Mef2c*, as well as myocardial components, such as *Troponin-I* and *Anf*, were down-regulated in *Smad4*-cKO hearts relative to control hearts. The expression of FHF markers, including *Hcn4*, *Tbx5*, and *Sfrp5*, was decreased, and the expression of SHF markers, such as *Tbx1* and *Islet1*, were unaffected or increased, in *Smad4*-cKO hearts relative to control hearts (Fig. 1B). Immunofluorescent staining of cTnT also revealed impaired cardiac differentiation in *Smad4*-cKO hearts (Fig. 1C). These observations suggested that Bmp signaling through Smad4 is required for proper cardiogenesis by regulating the expression of cardiac-specific transcription factors and/or cardiomyocyte-specific components.

### Smad4 regulates the expression and nuclear localization of Nkx2-5

**Figure 2:**
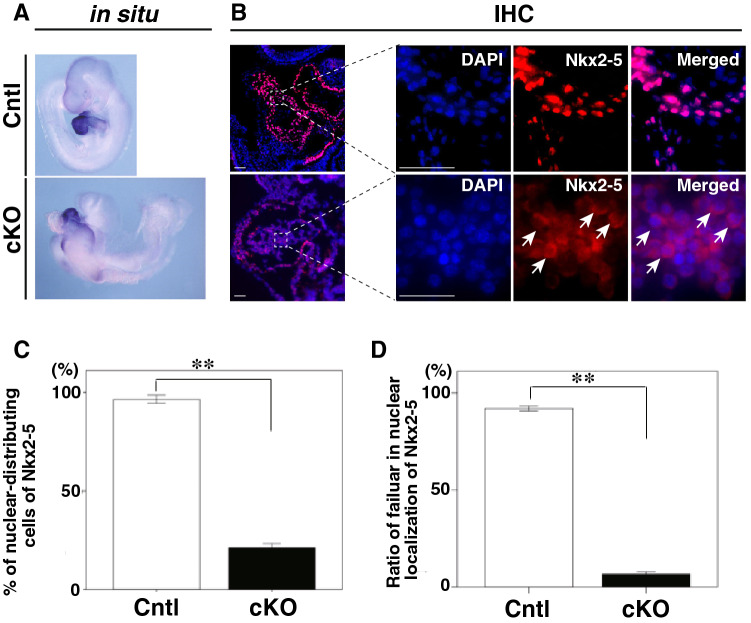
Lack of nuclear localization of Nkx2-5 in *Smad4*-cKO embryonic hearts. (**A**) Whole-mount ISH of control and *Smad4*-cKO embryos with a probe for *Nkx2-5* at E9.5. (**B**) Immunofluorescent staining of sagittal sections of control and *Smad4*-cKO embryos at E9.5 using anti-Nkx2-5 antibodies (red) and DAPI for detection of nuclei (blue). Arrows indicate the lack of nuclear localization of Nkx2-5. H, heart. Scale bar = 100 μm. (**C** and **D**) Percentage of Nkx2-5 protein distributing cell in control and *Smad4*-cKO embryonic hearts (**C**) and the percentage of cells showing nuclear localization of Nkx2-5 in control and *Smad4*-cKO embryonic hearts (**D**). Data were collected from 10 sections for each embryo (n = 3 and 3 for control and *Smad4*-cKO, respectively). Data are presented as mean ± SEM, **P < 0.01 (Mann–Whitney U test).

Since many genes encoding cardiac-specific transcription factors, including *Nkx2-5*, are down-regulated in *Smad4*-cKO hearts, and *Nkx2-5* has been suggested to be regulated by Bmp signaling through Smad-binding regions^22^, we hypothesized that lack of Nkx2-5 function might explain the impaired cardiac differentiation in *Smad4*-cKO embryos. In a previous study, Nkx2-5, a homeobox transcription factor, was reported to be a key factor in heart progenitor specification and differentiation by regulating the transcription of multiple cardiac-specific genes, including *Gja5* (Cx40), *Mef2c*, *Hey2*, *Nppa* (ANF), and *Isl1*^15^. Since Smad4 is required for *Nkx2-5* gene expression as it binds its upstream enhancer region *in vivo*^13^, we expected that *Nkx2-5* expression would be low or absent in *Smad4*-cKO hearts. However, *Nkx2-5* was expressed, albeit weakly (Fig. 1B), but detectable in *Smad4*-cKO hearts by in situ hybridization (ISH) (Fig. 2A). To confirm whether the Nkx2-5 protein was translated, immunofluorescent staining was performed and revealed that Nkx2-5 protein was weakly expressed (Fig. 2B, C). Interestingly, Nkx2-5 was present mainly in the nuclei of control heart cells, while they were found outside the nuclei of *Smad4*-cKO heart cells (Fig. 2B, D), suggesting that the loss of Smad4 prevented the nuclear localization of Nkx2-5.

### Impaired nuclear localization of Nkx2-5 in Smad4-cKO hearts is not a result of alterations in the phosphorylation status of Nkx2-5

The nuclear translocation of a transcription factor is often determined by the phosphorylation state of its NLS domain. Nkx2-5 contains a highly conserved NLS domain, and phosphorylation of serine 163 (Ser163) by Casein kinase 2 (CK2), a ubiquitously expressed and constitutively active Ser/Thr protein kinase^23^, is required for nuclear translocation. On this basis, we hypothesized that CK2 expression could be induced by Bmp signaling through Smad4 and examined this by qPCR and in situ hybridization. The expression level of *CK2α* was decreased, but clearly present, in *Smad4*-cKO hearts relative to control hearts (Supplementary Fig. S2A, S2B). We used *Csnk2a1* as a probe for the CK2 gene because the CK2 catalytic subunit α, rather than its other regulatory subunits, is expressed dominantly in the mouse embryonic heart^24^. Moreover, luciferase assays showed no activation of the upstream region of the *CK2α* gene upon stimulation of Bmp signaling (Supplementary Fig. S2C, S2D), suggesting that CK2 expression was not mainly regulated by Smad4.

We next determined whether excessive dephosphorylation of Nkx2-5 by the β-isoform of protein phosphatase 1 (PP1β) might explain the impaired nuclear localization of Nkx2-5. Since the regulatory subunit of PP1β, myosin phosphatase targeting subunit 1 (Mypt1), has been reported to be responsible for the nuclear exclusion of Nkx2-5 through direct interaction^25^, we expected Mypt1 to be dominantly localized in the nuclei and thereby prevent Nkx2-5 nuclear localization. However, in both control and *Smad4*-cKO hearts, Mypt1 was robustly expressed outside the nuclei (Supplementary Fig. S3). We also tested the possibility that the expression of protein phosphatase 1 regulatory subunit 3c (*Ppp1r3c*), a regulatory subunit of PP1 specifically expressed in the heart, might be increased in *Smad4*-cKO hearts. Here too, no difference was noted in the level of expression of *Ppp1r3c* by RT-PCR and ISH in *Smad4*-cKO and control hearts (Supplementary Fig. S4A, S4B), suggesting that Smad4 is not essential for *Ppp1r3c* expression. Taken together, these findings suggest that the phosphorylation state of the Nkx2-5 NLS might not be regulated by Bmp/Smad4 signaling.

### Smad4 regulates Nkx2-5 nuclear localization via protein interaction

**Figure 3:**
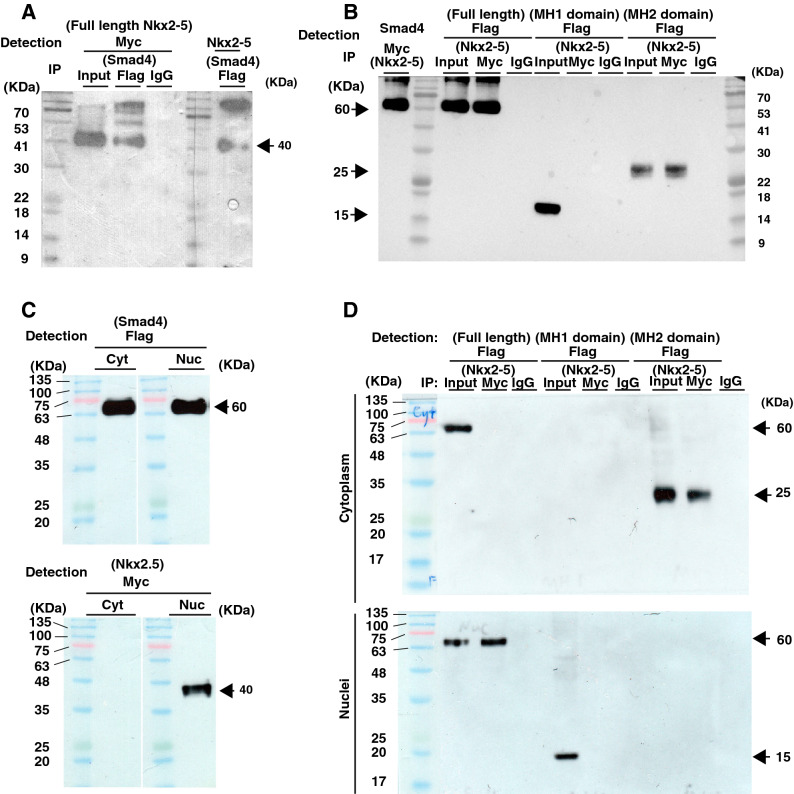
Interaction between Nkx2-5 and Smad4 via the MH2 domain. **(A**) Myc-tagged Nkx2-5 was co-immunoprecipitated with Flag-tagged Smad4 and detected using anti-Nkx2-5- and anti-Myc-antibodies. (**B**) Flag-tagged Smad4, and MH1- and MH2- deleted versions, were co-immunoprecipitated with Myc-tagged Nkx2-5 and detected using anti-Smad4 and Flag-antibodies. Nkx2-5 interacts with the Flag-tagged MH2 domain but not the MH1 domain. (**C**) Subcellular distribution of Flag-tagged Smad4 (upper) and Myc-tagged Nkx2-5 (lower) was determined by western blotting for extracts subcellular fractionated from cytoplasm or nuclei. (**D**) Interaction of Myc-Nkx2-5 to Flag-Smad4**-**full length, -MH1, or -MH2 is tested by Co-immunoprecipitation of extracts subcellular fractionated from cytoplasm or nuclei.

Since phosphorylated Smad1/5/8 forms a complex with Smad4 to translocate into the nucleus and regulate the transcription of target genes, we hypothesize that a protein complex containing both Smad4 and Nkx2-5 might similarly be involved in the proper nuclear translocation of Nkx2-5. Immunoprecipitation (IP) experiments using COS-7 cells demonstrated an interaction between Smad4 and Nkx2-5 (Fig. 3A, B).

Smad4 has two functional domains, MH1 and MH2, both of which bind to distinct proteins, such as importins and exportins. To gain insight into whether Nkx2-5 binds to either of these domains, we performed additional IP experiments with MH1- or MH2-deleted Smad4. As shown in Fig. 3B, the MH2 domain of Smad4 is responsible for binding to Nkx2-5 (Fig. 3B). These results indicate that Smad4 interacts with the MH-2 domain of Nkx2-5 to regulate Nkx2-5 nuclear translocation.

We then tested subcellular localization of Smad4 and Nkx2-5 by western blot analysis of subfractionated cellular compartments of COS-7 cells, which were transfected with their expression vectors (Fig. 3C). Smad4 was found in both cytoplasm and nucleus, while Nkx2-5 was detected only in the nucleus, suggesting that nuclear distribution of Nkx2-5 is occurred not only in cardiomyocytes but also in the fibroblasts. This observation gave rise to a question if the distribution of Nkx2-5 is controlled by Smad4. We performed IP experiments with subcellular compartments (Fig. 3D). We found that Nkx2-5 forms a complex with full length of Smad4 in nuclei or MH2 domain in cytoplasm, suggesting that interaction of full length of Smad4, including MH1 domain for translocation and MH2 domain for binding to Nkx2-5, is required for nuclear localization.

### Smad4 is essential for the nuclear localization of Nkx2-5

**Figure 4:**
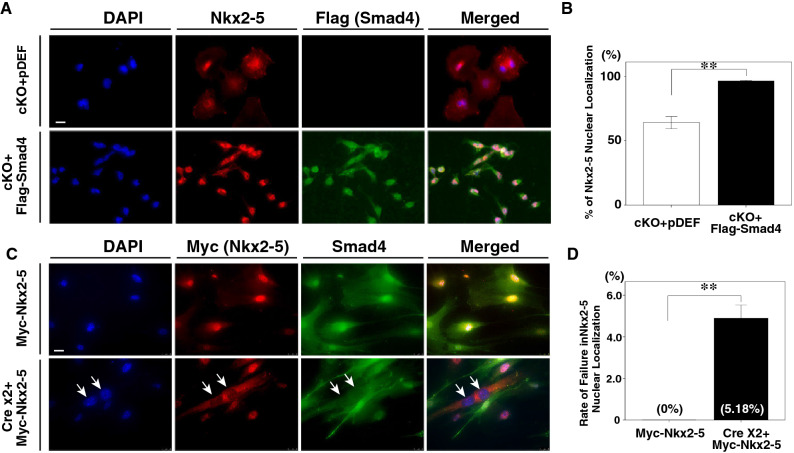
Introduction of *Smad4* rescues the nuclear localization of Nkx2-5 in mutant cells and depletion of the *Smad4* gene blocks the nuclear translocation of Nkx2-5 in fibroblast cells. (**A**) Double immunohistochemistry with anti-Nkx2-5 (red) and anti-Flag (green) antibodies. Cells from *Smad4*-cKO embryonic hearts were transfected with an empty vector (pDEF) as a control (upper panels) or Flag-tagged Smad4 expression vector (lower panels). Arrows show the failure of Nkx2-5 to localize to the nucleus. DAPI was used to stain nuclei. (**B**) Percentage of cells showing nuclear localization of Nkx2-5. Nuclear localization of Nkx2-5 was significantly increased in the presence of Smad4, compared to cells transfected with empty vector. Data were collected from 20 slides for each experiment (n = 4 and 4 for control and *Smad4*-cKO, respectively). Data are presented as mean ± SEM. *P < 0.01 (Mann–Whitney U test). (**C**) Double immunohistochemistry with anti-Myc (red) and anti-Smad4 (green) antibodies. Fibroblast cells from *Smad4f.*^*/f*^ embryos were transfected with the Myc-Nkx2-5 expression vector and showed nuclear localization of Nkx2-5. After depleting Smad4 by transfecting fibroblasts with Cre expression vector twice, Nkx2-5 was no longer localized to the nucleus. DAPI was used to stain nuclei. (**D**) The proportion of non-nuclear localization of Nkx2-5 in control (transfected with Myc-Nkx2-5) and *Smad4*-cKO (transfected with Myc-Nkx2-5 after two rounds of Cre expression vector transfection) cells. Data were collected from 20 slides for each experiment (n = 3 and 3 for control and *Smad4*-cKO, respectively). Data are presented as mean ± SEM. **P < 0.01 (Mann–Whitney U test).

We further examined whether the introduction of Smad4 would be sufficient to restore the nuclear localization of Nkx2-5 by immunohistochemical analysis. *Smad4*-cKO heart cells, which express exogenous Flag-tagged Smad4 showed a significant increase in the ratio of cells with Nkx2-5 localized in the nucleus (Fig. 4A, B), suggesting that Smad4 is required for the nuclear localization of Nkx2-5.

Since our results indicate that Smad4 seems required for the nuclear translocation of Nkx2-5, we next tested whether exogenous Nkx2-5 would localize to the nucleus of not only cardiomyocytes but also in other types of cells that express CK2 and Smad4, such as fibroblasts. In primary cultures of fibroblasts derived from *Smad4f.*^*/f*^ embryos transfected with an expression vector for *Nkx2-5*, Nkx2-5 was able to enter the nucleus. This suggests that the nuclear translocation of Nkx2-5 is not limited to a specific cell type. We also tested whether Smad4 is required for the nuclear translocation of Nkx2-5. After transfecting fibroblasts derived from *Smad4f.*^*/f*^ embryos with a Cre expression vector to abolish Smad4 expression, the cells were further transfected with an expression vector for *Nkx2-5*. As shown in Fig. 4C, D, Nkx2-5 failed to enter the nucleus in the absence of Smad4 expression, suggesting that Smad4 is required for the nuclear localization of the Nkx2-5.

## Discussion

In this study, we successfully generated mice that have a deletion of the *Smad4* gene in cardiac progenitor cells by crossing *Sfrp5*-Cre mice with *Smad4f.*^*/f*^ mice. These mice showed hypoplastic heart tubes with disruption of cardiomyocyte differentiation, suggesting that Smad4 is involved in cardiac development. Bmp/Smad4 signaling has been suggested to regulate the transcription of cardiac-specific transcription factors^8^. We found that expression of such transcription factors, especially those related to the FHF but not SHF, were down-regulated in *Smad4-*cKO hearts (Fig. 1B). This suggests that disruption of the *Smad4* gene in *Sfrp5*-expressing cells, which are predominantly cardiac progenitor cells for the FHF, resulted in the downregulation of cardiac-specific genes and may explain the hypoplastic heart tubes observed in *Smad4-*cKO embryos.

*Nkx2-5* transcription was not completely eliminated in *Smad4*-cKO hearts, suggesting that Bmp signaling through Smad4 is likely not required for the expression of *Nkx2-5*. Since the heart tube itself was formed in *Smad4*-cKO embryos, Bmp/Smad4 does not appear to be required for cardiac cell fate decisions. One possible explanation is that the disruption of *Smad4* was incomplete and residual Smad4 could have activated the transcription of *Nkx2-5*. However, *Sfrp5* is expressed in cardiac development before the *Nkx2-5* gene, and *Smad4* disruption in the early epiblast using *Sox2-*Cre results in rudimentary heart formation and the expression of cardiac-specific markers, including *Nkx2-5*^26^*.* Moreover, the loss of type1-Bmp receptor in early mesoderm progenitors using *Mesp1*-Cre also results in the formation of a hypoplastic heart^7^. Thus, Bmp/Smad4 signaling might not be a trigger for initial cardiac cell specification.

**Figure 5:**
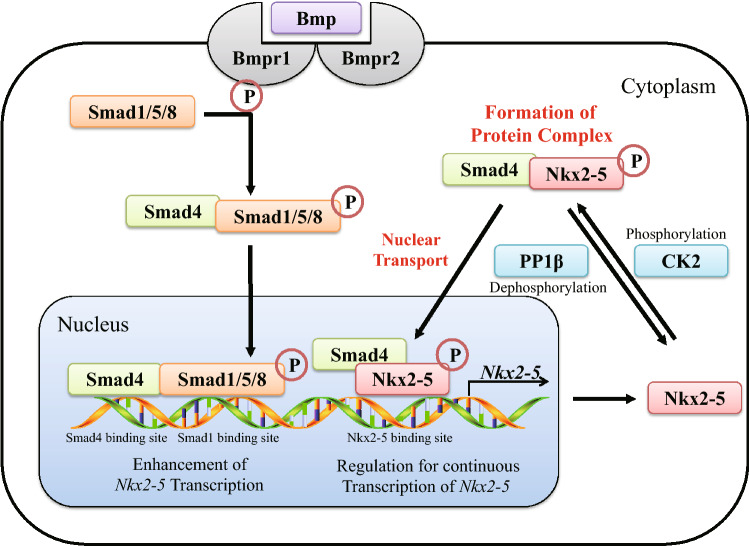
Schematic of Smad4 regulation of cardiac-specific gene expression and nuclear localization of Nkx2-5. Bmp activates its receptor, which then phosphorylates the Smad1/5/8 complex. In turn, the Smad1/5/8 complex binds to Smad4, which subsequently translocates into the nucleus to enhance *Nkx2-5* transcription. Nkx2-5 protein is phosphorylated by CK2 and forms a protein complex with Smad4. This complex translocates into the nucleus and regulates *Nkx2-5* transcription, thereby maintaining Nkx2-5 expression. Through this mechanism, Smad4 plays a decisive role in cardiac development and myocardial differentiation.

Disruption of Smad4 resulted in the failure of Nkx2-5 to localize to the nucleus (Fig. 5) in both cardiomyocytes and fibroblasts. The NLS sequence in the homeobox of Nkx2-5 is required for nuclear translocation, which is controlled by regulating the phosphorylation state of a serine residue near the NLS by CK2 and PP1β^23,25^. In our experiments, however, the expression of CK2 and PP1β were not transcriptionally controlled by Bmp/Smad4. Since CK2 and PP1β are extensively involved in fundamental metabolic processes, they may be constitutively expressed ubiquitously to serve as housekeeping genes. This suggests that CK2 is likely always in a position to phosphorylate Nkx2-5, implying that phosphorylation of Nkx2-5 is unlikely to be involved in cardiomyocyte fate decisions. Moreover, Smad4 is ubiquitously expressed after the early definitive endoderm and mesoderm are formed^27^, suggesting that the nuclear localization of Nkx2-5 should be inevitable once Smad4 is synthesized. Consequently, nuclear localization might be a prerequisite for transcription factors, such as Nkx2-5, to play their respective roles in cardiac differentiation.

Nkx2-5 interacted with the MH2 domain of Smad4. Smad4 is known to bind to nuclear transporter proteins, such as Importin, via the MH1 domain, and bind to Exportin (Chromosome Region Maintenance 1; CRM1) via its linker region^28–33^. The MH2 domain, however, is implicated in transcriptional activation and interactions with other Smad proteins to form homo- and heteromeric complexes. This suggests that Nkx2-5 could behave similarly to other Smad proteins with regard to its nuclear localization (Fig. 5). In the nucleus, we speculate that the interaction between Smad4 and Nkx2-5 persists and allows for the transcriptional regulation of genes involved in heart development. For instance, Nkx2-5 regulates its own continuous expression^13,34^. Moreover, GATA4 reportedly binds to an upstream region of the *Nkx2-5* transcriptional start site and controls *Nkx2-5* expression in cooperation with Smad1/4^22^. Some studies have also reported an interaction between GATA4 and Nkx2-5^35,36^. Thus, after the Smad4-Nkx2-5 complex translocates into the nucleus, a complex containing Smad4, Nkx2-5, and GATA4 may allow for the sustained expression of Nkx2-5, thereby acting as a maintenance mechanism for mature cardiomyocytes.

Our results provide evidence that Smad4 is required for the transcription of several cardiac-specific genes, despite the fact that initial tube formation occurs and residual Nkx2-5 expression remains in *Smad4*-cKO embryonic hearts. This suggests that while Bmp/Smad signaling does not serve as a trigger for myocardial differentiation, it can promote cardiac differentiation by controlling the nuclear localization of Nkx2-5 via Smad4. Hence, Smad4 could function not only as a mediator of Bmp signaling for the transcriptional regulation of cardiac-specific genes but also as a regulator of the nuclear localization of Nkx2-5 to ensure proper cardiac differentiation.

## Methods

### Mice

The *Sfrp5 *^*Cre/*+^*; Smad4 *^*del/del*^ mice with a C57Bl6 background were obtained by crossing *Sfrp5 *^*Cre/Cre*^*; Smad4 *^*del/*+^ mice with *Smad4 *^*floxed/floxed*^ mice, which carry the *Smad4* gene flanked by loxP recognition sites. Generation of the *Sfrp5 *^*Cre/*+^ line was described previously^21^. The *Smad4 *^*floxed/floxed*^ line was a gift from Dr. CX Deng^37^. All procedures had local approval by the research ethics committee of Hiroshima University (A18-109–2) and conformed to the NIH Guide for the Care and Use of Laboratory Animals and all study carried out in compliance with the ARRIVE guidelines. Mouse tissues for all experiments were isolated after euthanizing mice by CO_2_ asphyxiation.

### Ex vivo* rescue experiment*

Hearts were dissected from *Smad4*-cKO embryos at E9.5. Cardiomyocytes were harvested by trypsinization with 0.1% (w/v) Trypsin (Nacalai Tesque, Kyoto, Japan, 32778-05) for 2 min, seeded onto 24 mm × 24 mm glass plates, and cultured in 6-well tissue culture plates. A plasmid containing *Smad4* cDNA (Flag-tagged) or an empty vector (pDEF) was transfected using 3.23% (w/v) Polyethylenimine (PEI Max, Cosmobio, Tokyo, Japan, 24765-2) on the following day. The immunofluorescent analysis was performed 24 h after transfection to detect the cellular distribution of Nkx2-5.

Fibroblast cells were obtained from *Smad4f.*^*/f*^ embryos using the same method described above and cultured in 6-well tissue culture plates. To delete *Smad4*, plasmids containing *Cre* cDNA were transfected into the fibroblasts twice using 3.23% (w/v) PEI.

### Cell culture

COS-7 cells were maintained in DMEM (Life Technologies, Carlsbad, CA, 11995-065) containing 4.5 g/L D-glucose supplemented with 1 mM sodium pyruvate, 4 mM L-Glutamine, and 10% (v/v) fetal bovine serum (CELLect, Kefar Sava, Israel, 2916754) at 37 °C and 5% CO_2_.

### Transfection

COS-7 and fibroblast cells were transfected with expression plasmids, including Flag-tagged *Smad4*, *MH1*, or *MH2* (a gift from Dr. T. Imamura, Ehime University) and Myc*-*tagged *Nkx2-5* (a gift from Dr. Koshiba, Toyo University) using 3.23% (w/v) polyethyleneimine (PEI Max, Cosmobio, Tokyo, Japan, 24765-2) following the same steps described above for the rescue experiment.

### *Whole-mount *in situ* hybridization*

Whole-mount in situ hybridization was performed as described previously^21^. Embryos were dissected at E9.5 and fixed with 4% paraformaldehyde in PBS for durations of 1 h to overnight. After dehydration using a methanol series, embryos were rehydrated with 0.1% Tween 20 in PBS (PBST), bleached with 6% H_2_O_2_ in PBS, treated with Protease K, and then hybridized with digoxygenin (DIG)-labeled probes at 68ºC overnight. Embryos were then washed with 5 × and 2 × SSC/50% formamide and PBST. To detect DIG-labelled probes, embryos were incubated with anti-DIG antibodies after treatment with blocking reagent for 1 h, and the signal was developed with BM purple AP substrate (Roche, Basel, Switzerland).

### Real-time PCR

Real-time PCR was performed as previously described^21^. Total RNA was isolated using TRIzol (Invitrogen, Carlsbad, CA). Reverse transcription was performed with the ReverTra Ace qPCR RT Kit (TOYOBO, Osaka, Japan). Real-time PCR was conducted using SYBR Premix Ex Taq II (Kapa Biosystems Inc., Wilmington, MA). Intensities of PCR products were measured and analyzed using Mini-Opticon (Bio-Rad, Hercules, CA). Amplification conditions were: 5 s at 95 °C, 20 s at 60 °C, and 15 s at 72 °C for 49 cycles. *G3pdh* was amplified as an internal control. Primers used in the present study are shown in Supplementary Table S1.

### Immunohistochemistry

Embryos were embedded in optimum cutting temperature (OCT) compound (Sakura Tissue-Tek, Tokyo, Japan, 4583) after fixation and cut into 10–14 μm sections. After washing with PBS and blocking with 1% BSA in PBS for 1 h, sections were incubated with 500 × diluted primary antibodies, including anti-Smad4 (Santa Cruz, Dallas, TX, sc-7966), anti-Mypt1 (Proteintech, Rosemont, IL, 22117-1-AP), anti-Flag (DYKDDDDK)-Tag (Sigma Aldrich, St. Louis, MO, F3165 or Cell Signaling Technology, Danvers, MA, 8146S), anti-Myc Tag (Cell Signaling Technology 2278S), anti-Nkx2-5 (Abcam, Cambridge, UK, ab35842 or ab97355), and anti-TnT (Thermo, Waltham, MA, MS-295-P0) at 4 °C overnight. Sections were then incubated with 500 × diluted secondary antibodies conjugated with Alexa Fluor 488 or 555 (Molecular Probes, Eugene, OR). Nuclei were stained with 4, 6-diamidino-2-phenylindole (FUJIFILM Wako Pure Chemical Corporation, Osaka, Japan).

### Nuclear and cytoplasmic protein extraction

COS-7 cells, transfected expression vectors, were collected after 24 h, and then their nuclei and cytoplasm fractions were separated. Their containing proteins were harvested by using a NE-PER Nuclear and Cytoplasmic Extraction Reagents (Thermo Fisher Scientific, Waltham, MA, 78833) following the instruction protocols and stored at – 80 °C for further use.

### Western blotting

Cultured cells were washed with cold PBS and lysed on ice with Sample buffer (100 mM DTT, 50 mM Tris/HCl pH 8.0, and 0.1% (w/v) SDS). After centrifugation at 15,500 rpm for 10 min at 4 °C, supernatant fractions were collected and analyzed by SDS-PAGE and immunoblotting.

### Immunoprecipitation

Cultured cells were washed with cold PBS and lysed on ice with RIPA buffer (150 mM NaCl, 5 mM EDTA pH 8.0, 50 mM Tris/HCl pH 8.0, 1% (v/v) IGEPAL CA-630, 0.1% (w/v) SDS, and 0.5% (w/v) sodium deoxycholate) containing phenylmethylsulfonyl fluoride (PMSF, Tocris Bioscience, Bristol, UK) for 30 min. After centrifugation at 15,000 rpm for 10 min at 4 °C, supernatants were collected and used for immunoprecipitation (IP). IP was performed with the Immunoprecipitation Starter Pack (GE Healthcare, Chicago, IL) according to the manufacturer’s instructions, using 100 μl column resin conjugated with 5 μg anti-Flag antibody, 5 μg anti-Myc antibody, 5 μg anti-Smad4 antibody, 5 μg anti-Nkx2-5 antibody, or 100 μg IgG, and then analyzed by immunoblotting after SDS-PAGE. Signal detection was performed with the LuminoGraphI (ATTO, Amherst, NY, WSE-6100H-CSP) system.

### Statistical analysis

Data are expressed as mean ± SEM and were analyzed using one-way analysis of variance (ANOVA) with the Tukey–Kramer post hoc test for multiple comparisons or the nonparametric Mann–Whitney U test to compare two independent populations. Statistical analyses were performed using SPSS 20.0 software (IBM Corp. Released 2011. IBM SPSS Statistics for Windows, Version 20.0. Armonk, NY: IBM Corp. https://www.ibm.com/analytics/spss-statistics-software). *P < 0.05 was considered statistically significant.

## Supplementary Information

Supplementary Information.
