# Supplementary material for: Smad4 regulates the nuclear translocation of Nkx2-5 in cardiac differentiation
Source: Sci Rep. 2021 Feb 11;11:3588. doi: 10.1038/s41598-021-82954-2 (PMC7878807; doi:10.1038/s41598-021-82954-2)

Title: **Smad4 regulates the nuclear translocation of Nkx2-5 in cardiac differentiation**

Author names: Wenyu Hu[1,2], Anqi Dong[1], Kohei Karasaki[1], Shota Sogabe[1], Daiki Okamoto[1], Masato Saigo[1], Mari Ishida[1], †Masao Yoshizumi[1], and †Hiroki Kokubo[1]

Affiliations:[1]Department of Cardiovascular Physiology and Medicine, Graduate School of Biomedical and Health Sciences, Hiroshima University, 1-2-3 Kasumi, Minamiku, Hiroshima 734-8551, Japan

[2]Department of Cardiology, the First Affiliated Hospital of China Medical University, Shenyang, Liaoning 110001, China

†For co-correspondence: Masao Yoshizumi and Hiroki Kokubo

yoshizumi@umin.net and hkokubo@hiroshima-u.ac.jp

Short title: **Smad4 regulates the nuclear localization of Nkx2-5**

Supplemental Table S1. Primers used for Real-time PCR

	Forward	Reverse
Csnk2a1	5’-ACACTGTTGTGAAGGACCAGG-3’	5’-CATCAGAAGGTAGATGGGGGT-3’
Ppp1r3c	5’-GCTAATGGGAGGATCTTCTGG-3’	5’-GGTCAAGTTCTCCACTCTCCC-3’
Nkx2-5	5’-GCTACAAGTGCAAGCGACAG-3’	5’-GGGTAGGCGTTGTAGCCATA-3’
Hcn4	5’-CTCAACTCAGGCGTCTTCAAC-3’	5’-CCACCGAAGTAGTAGCAGCAG-3’
Mef2c	5’-GTACACCGAGTACAACGAGC-3’	5’-CCTGTGTTACCTGCACTTGG-3’
Gata4	5’-TCTCACTATGGGCACAGCAG-3’	5’-CGAGCAGGAATTTGAAGAGG-3’
Nppa	5’-GAAGCAGCTGGATCTTCGTAG-3’	5’-TGTGTACAGTGCGGTGTCCAA-3’
Tnni2	5’-CCAGCACTGCTGCACAGCA-3’	5’-AGACATGGAGCCTGGGATG-3’
Hey1	5’-ACTTCCCCAGGGAATGTGTC-3’	5’-ATCGGAGTTTGGGGTTTCGG-3’
Hey2	5’-GCAGCAGTGATGACATCCTC-3’	5’-GCAGAATCTGCATGGGCAAAC-3’
Gja5	5’-TTCATCGTAGGCCAGTACCTC-3’	5’-CAGGTGGTAGAGTTCAGCCAG-3’
Gja1	5’-CTGTACTTGGCTCAGGTGTTC-3’	5’-CACCTCTCATCTTCACCTTGC-3’
Tbx5	5’-CTTCCCTACCAGCACTTCTCC-3’	5’-GTGTAGAGAAACTCTGGGGGC-3’
Sfrp5	5’-TGTGCTCCAGTGACTTTGTGG-3’	5’-TCAGGTTGTCTAACTGTGGGC-3’
Isl1	5’-CACGACCAGTATATTCTGAGGG-3’	5’-TCTCTCCACCACATCGTGGTC-3’
Tbx1	5’-ACACCTGGCCGAGTACACTAC-3’	5’-ACTGTCTTTTCGAGGGTCCAC-3’
Tbx2	5’-CCTGCTAATGGACATCGTGGC-3’	5’-CAAGATGTCATTGGCTCGCAC-3’
Tbx3	5’-CATCGTCAGAGCCAACGATATC-3’	5’-CCTCAAACACTCTCATGGAC-3’
Bmp2	5’-CCAGGTTAGTGACTCAGAACAC-3’	5’-TCATCTTGGTGCAAAGACCTGC-3’
G3pdh	5’-ACCACAGTCCATGCCATCAC-3’	5’-TCCACCACCCTGTTGCTGTA -3’

Supplementary information

Supplementary Figure S1. Morphology of *Smad4*-cKO and littermate control embryos.

Compared to the littermate control (*Sfrp5* $^{Cre/+}$; *Smad4*$^{del/+}$), *Smad4*-cKO (*Sfrp5* $^{Cre/+}$; *Smad4* $^{del/del}$) hypoplastic heart tubes were formed with a clearly flawed looping shape in the severe phenotypes. Scale bar=200 µm.

Supplementary Figure S2. *CK2* may not be a direct downstream target of Smad4.

(A) Relative expression of *CK2* in control and mutant embryonic hearts at E9.5 (n=9 and 7 for littermate control and *Smad4*-cKO, respectively). Data are presented as mean±SEM. **P*<0.05 (Mann-Whitney U test). (B) Whole-mount ISH analysis using a *CK2* antisense probe in control and *Smad4*-cKO embryos at E9.5. (C) Schematic of reporter plasmids used in the luciferase assay. The genomic region of the *CK2* gene, which contains prospective Smad4-binding sites upstream of the transcription starting site of *CK2,* was inserted into the Luc reporter vector, pGL-basic (*CK2* -Pst1-Luc and *CK2* -EcoR1-Luc). (D) Luciferase assay. In order to clarify the relationship between Bmp signaling and *CK2*, Alk6KR (dominant-negative form), Alk6QD (activated form), Smad5, and/or empty vector (pDEF) were transfected together with reporter plasmids into NIH3T3 cells as indicated. Statistical analysis was performed by one-way ANOVA, with Tukey–Kramer *post hoc* analysis for multiple comparisons. n=3 for each group. Data are presented as mean±SEM.

Supplementary Figure S3. Mypt1 expression is not affected in *Smad4*-cKO hearts.

Immunofluorescent staining using anti-Mypt1 (green) antibody on sagittal sections of control and *Smad4* mutant embryos. DAPI was used to stain nuclei.

Supplementary Figure S4. Bmp signaling via Smad4 may not be essential for *Ppp1r3c* expression.

(A) Relative expression of *Ppp1r3c* in littermate control and *Smad4*-cKO embryonic hearts at E9.5. n=9 and 7 for control and *Smad4-cKO*, respectively. Data are presented as mean±SEM. (Mann-Whitney U test). (B) Whole-mount ISH with the probe for *Ppp1r3c*. Signal was detected in the hearts of control and *Smad4*-cKO embryos.

Supplementary Figure S5. Raw images at different exposure times for Figure 3A.

The immunoblotted membrane was contacted with a film in 5 min (A), 1 min (B), or 30 sec (C). Then the film was developed and scanned.

Supplementary Figure S6. Raw images at different exposure times for Figure 3B.

The immunoblotted membrane was directly scanned for 1 min (A), 5 min (B), 10 min (C), or 20 min (D).

Supplementary Figure S7. Raw images at different exposure times for Figure 3C.

The immunoblotted membrane was directly scanned for 30 sec (A), 1 min (B), 3 min (C), or 5 min (D).

Supplementary Figure S8. Raw images at different exposure times for Figure 3D.

The immunoblotted membrane was directly scanned for 1 min (A), 2 min (B), or 3 min (C).

Hu et al.
Supplemenatal figure 1

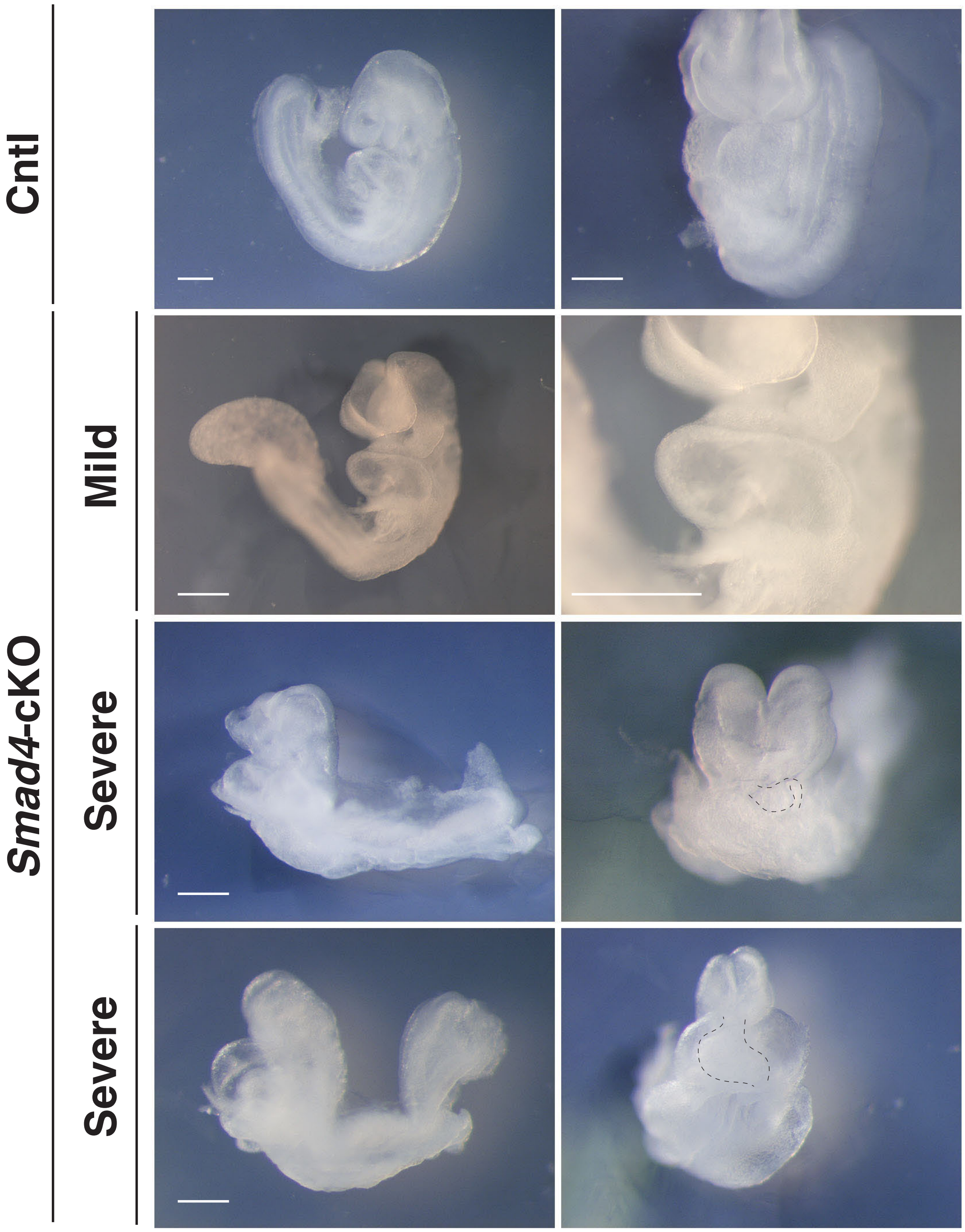

Hu et al.
Supplemenatal figure 2

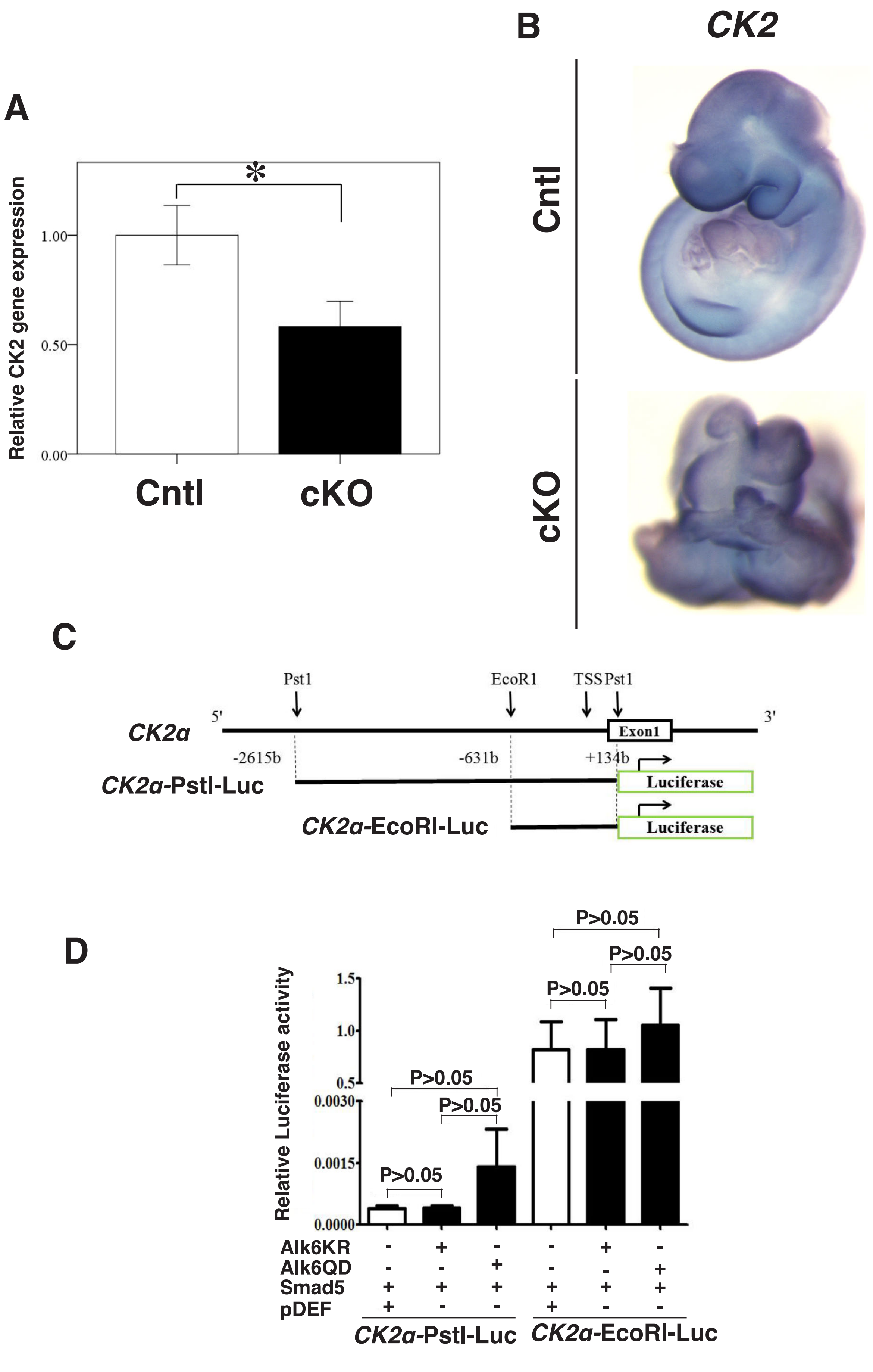

Hu et al.
Supplemenatal figure 3

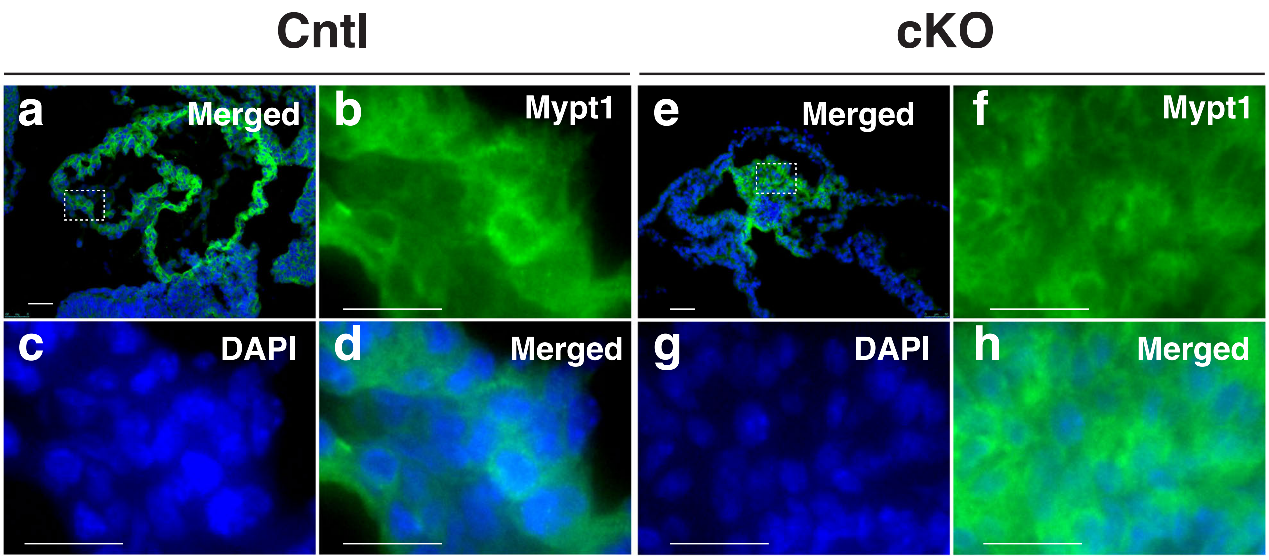

Hu et al.
Supplemenatal figure 4

A

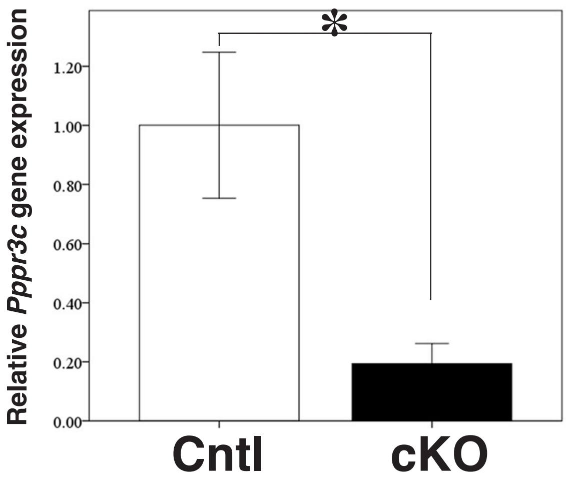

B

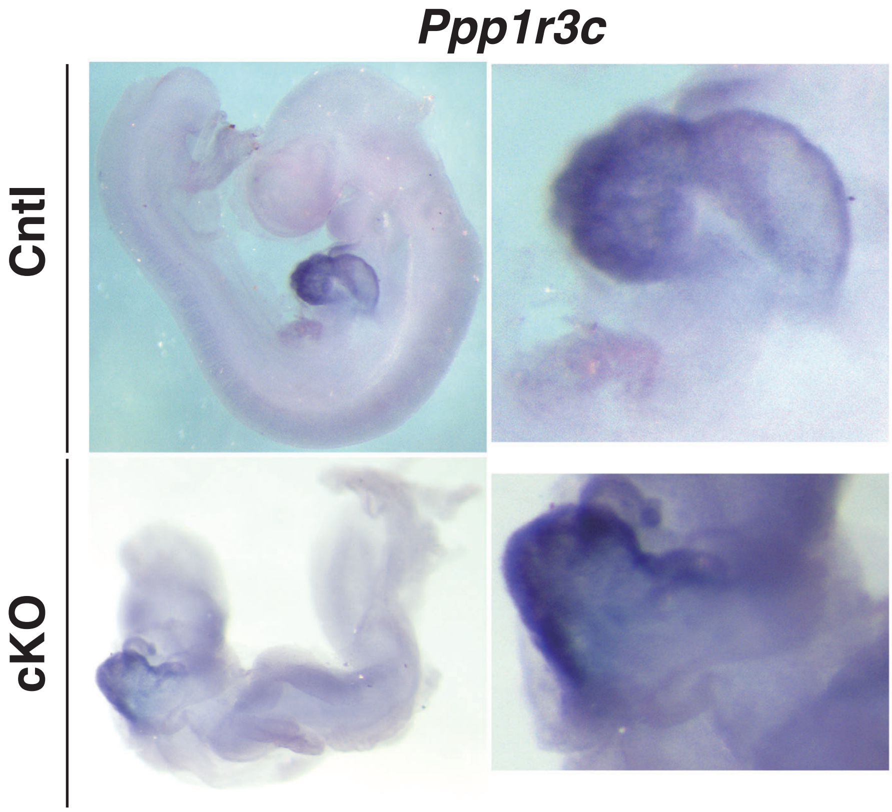

Hu et al.
Supplemenatal figure 5

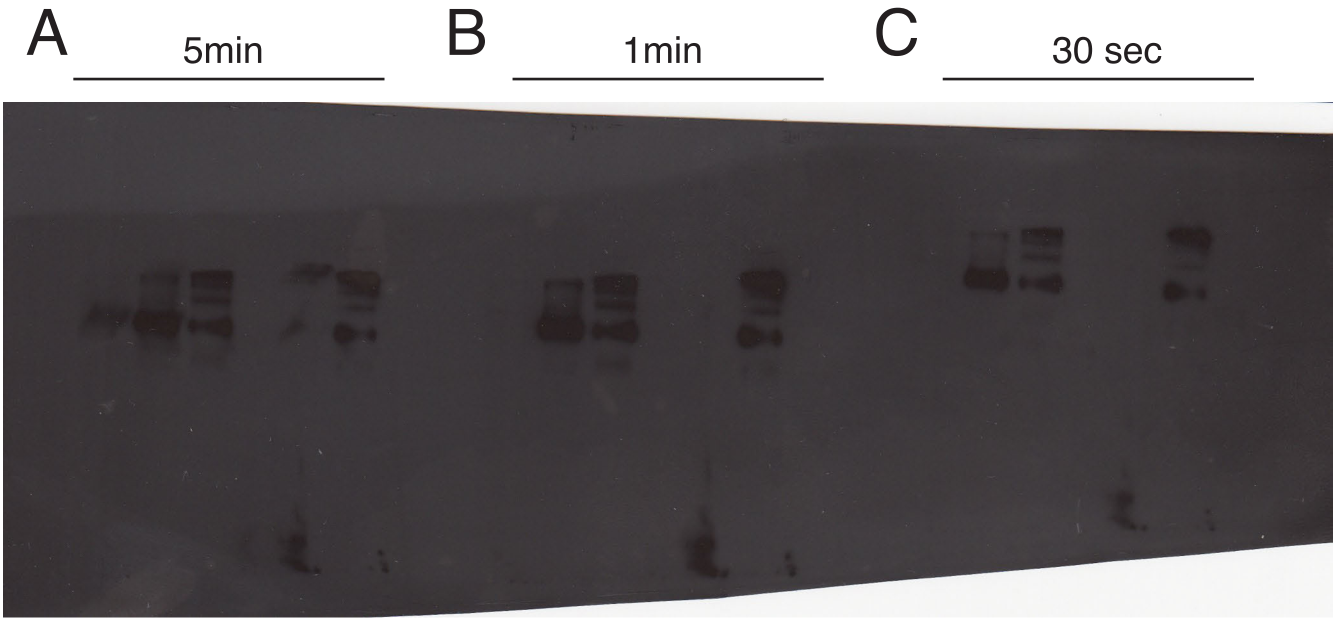

Hu et al.
Supplemenatal figure 6

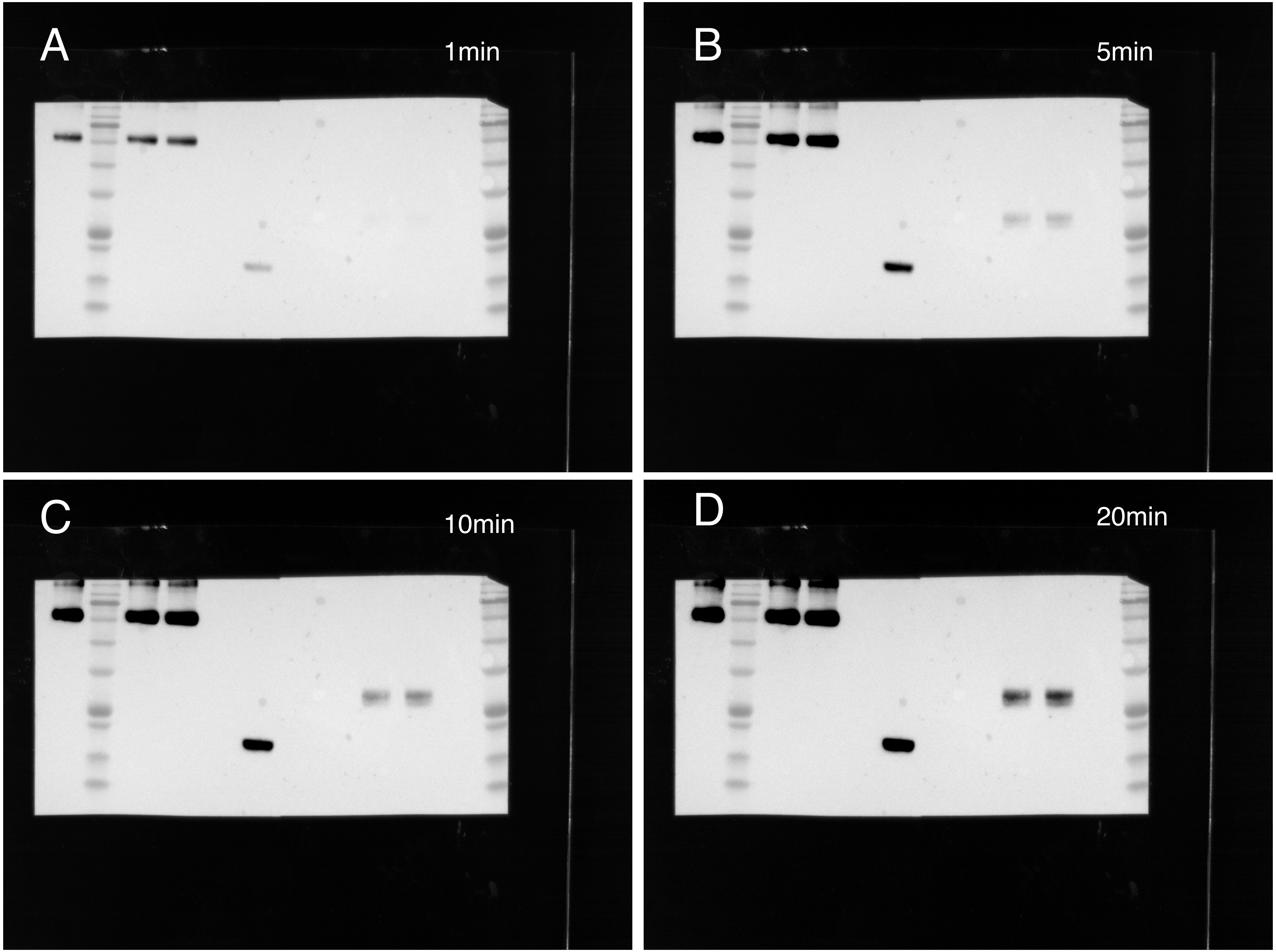

Hu et al.
Supplemenatal figure 7

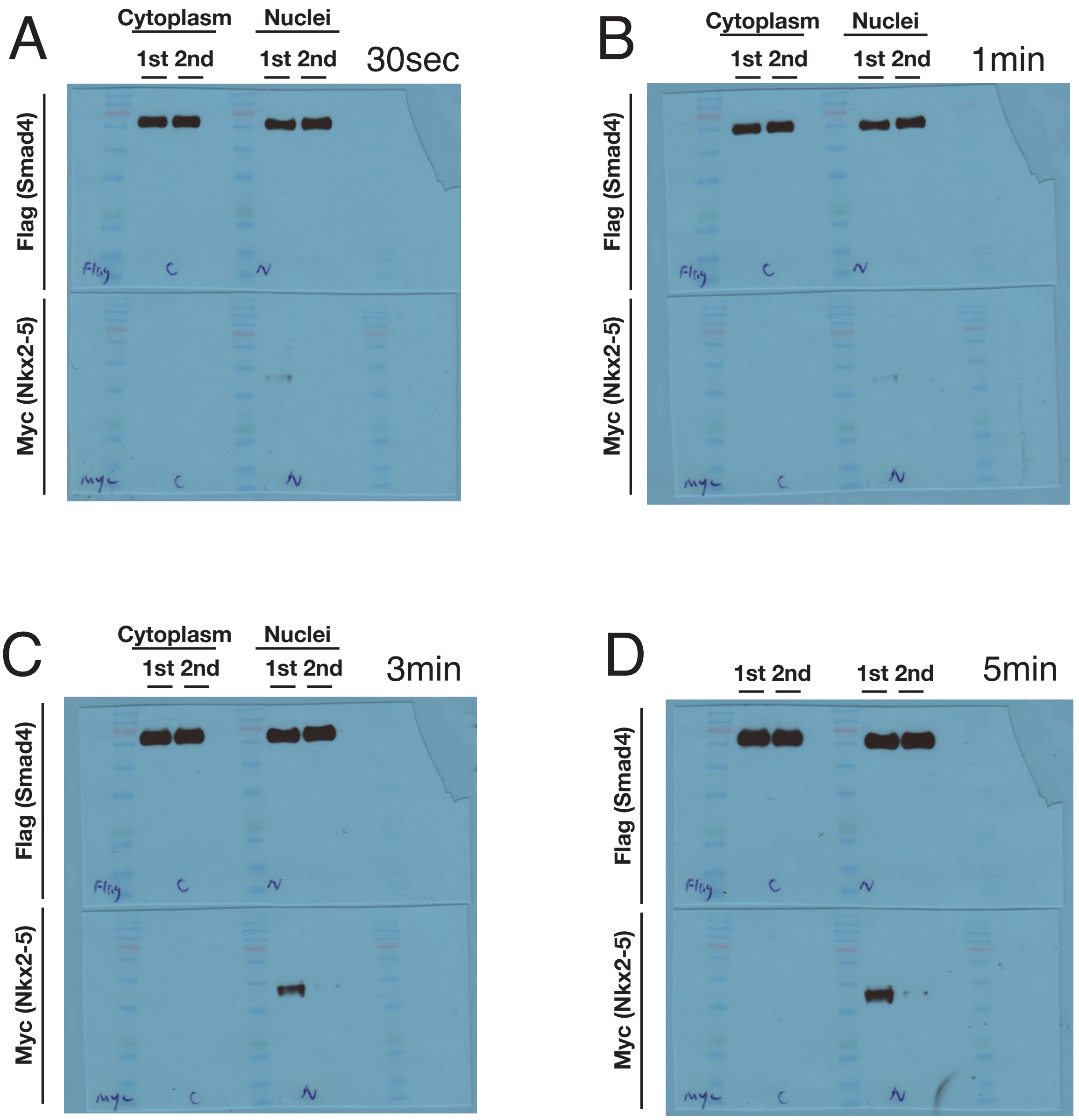

Hu et al.
Supplemenatal figure 8

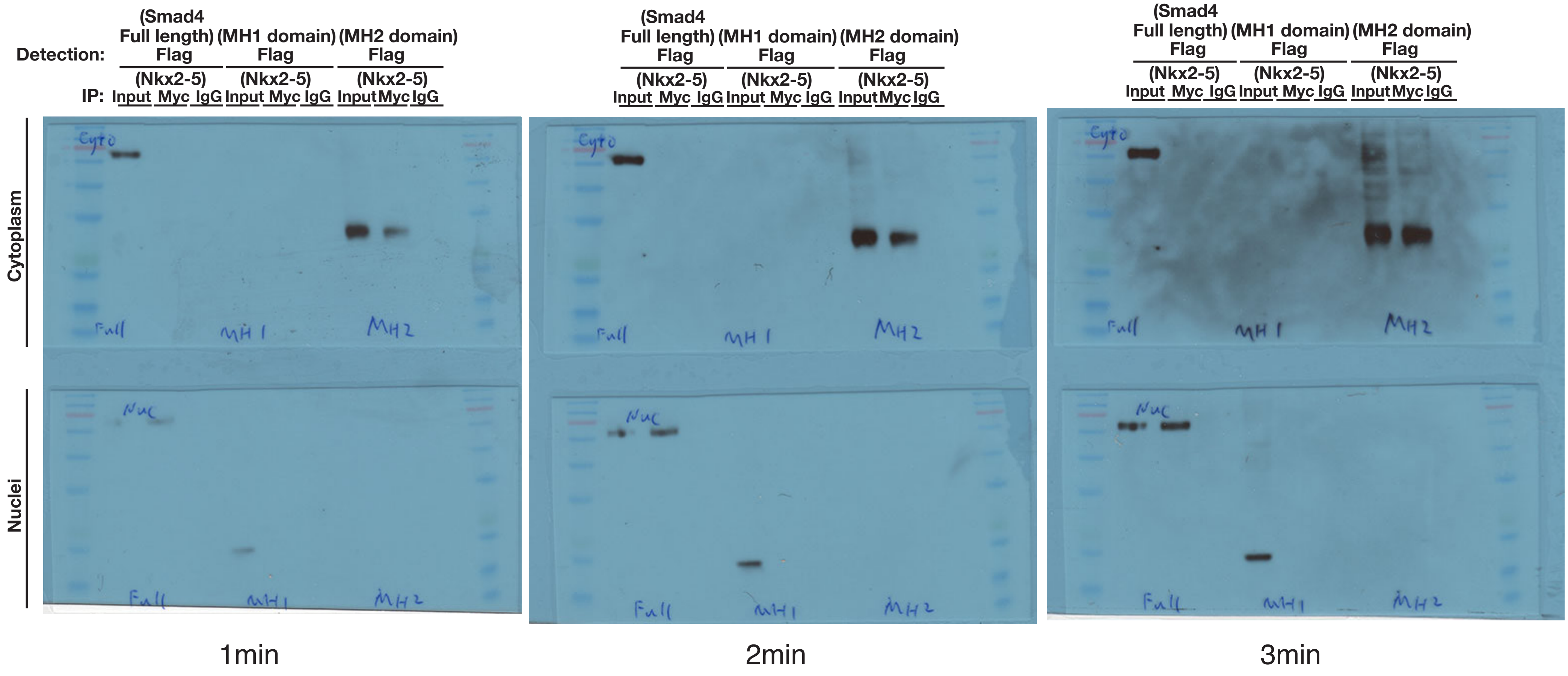

Supplement: Supplementary file 1 — Supplementary Information. [file 41598_2021_82954_MOESM1_ESM.pdf]
